# Effect of Exercise on Vascular Function and Blood Lipids in Postmenopausal Women: A Systematic Review and Network Meta-Analysis

**DOI:** 10.3390/ijerph191912074

**Published:** 2022-09-23

**Authors:** Chenxi Xin, Mingyi Ye, Qianqian Zhang, Hui He

**Affiliations:** 1 Department of Chinese Academy of Sport and Health, Beijing Sport University, Beijing 100084, China; 2 Department of Physical Education, Shanghai Jiao Tong University, Shanghai 200240, China

**Keywords:** exercise, blood lipids, vascular function, postmenopausal women, network meta-analysis

## Abstract

This study aimed to compare and rank the effectiveness of aerobic exercise (AE), resistance training (RT), combined training (CT), and water exercise (WE) on vascular function and blood lipids in postmenopausal women using a network meta-analysis (NMA). Methods: We searched the PubMed, Cochrane, Embase, Web of Science, and EBSCO (SPORTDiscus) databases to identify randomized controlled trials investigating the effects of exercise on vascular function and blood lipids in postmenopausal women. The retrieval period was from inception to March 2022. Two reviewers independently screened the retrieved articles, extracted pertinent data, and assessed the risk of bias of the included studies. Results: A total of 38 studies involving 1407 patients were included in this study. The results of the NMA indicated that WE had the greatest effect on systolic blood pressure (SBP) (surface under the cumulative ranking [SUCRA] = 84.9) and total cholesterol (TC) (SUCRA = 93.1); CT had the greatest effect on triglycerides (TG) (SUCRA = 96.2), high-density lipoprotein cholesterol (HDL-C) (SUCRA = 94.8), and diastolic blood pressure (DBP) (SUCRA = 91.1); RT had the greatest effect on low-density lipoprotein cholesterol (LDL-C) (SUCRA = 79.4). Conclusion: The results suggest that exercise can effectively improve the PWV, SBP, and DBP and the levels of TC, TG, and LDL-C in postmenopausal women. WE had the best effect on improving TC and SBP. CT had the best effect on improving TG, HDL-C, and DBP. To improve LDL-C, RT can achieve a good effect. Considering the limitations of NMA, more RCTS need to be performed in the future to provide more direct evidence of the effectiveness of various exercise interventions on vascular health in postmenopausal women.

## 1. Introduction

Cardiovascular disease (CVD) is the most lethal disease in the world [1]; 17.79 million people were reported to have died from CVD in 2016, accounting for 46.2% of all global deaths [2]. A prospective cohort study showed that more than 40% of CVD can be attributed to metabolic risk factors (e.g., abnormal lipid levels) [3]. In postmenopausal women, decreased estrogen makes patients more susceptible to many cardiovascular health problems, such as elevated blood pressure (BP), dyslipidemia, increased pulse wave velocity (PWV), and abnormal endothelial function [4,5,6]. In addition, a large number of studies have shown that the prevalence of CVD in men is higher than that in women before menopause, but, with the increase in women’s age and the rapid decline in estrogen levels in the body, the cardiovascular protective effect is weakened, resulting in the incidence of CVD in women after menopause being much higher than that in men of the same age [7,8,9]. Therefore, it is particularly important to prevent and delay the onset of CVD among postmenopausal women.

Studies have shown that more than half of the causes of CVD death are related to risk factors such as hypertension and dyslipidemia [10]. The coexistence of hypertension and dyslipidemia can accelerate the process of atherosclerosis and increase the risk of cardiovascular disease [11], and early identification of arterial lesions is helpful for early diagnosis. PWV is an early indicator that accurately reflects arterial vascular disease. Because it is simple, effective, economical, and non-invasive, PWV has become an important indicator for evaluating arterial stiffness [12]. Therefore, the simultaneous management of BP, blood lipids, and arterial stiffness is an important means to prevent cardiovascular diseases.

It is well known that physical activity, as well as a healthy lifestyle, can substantially reduce the incidence of CVD [13,14]. Currently, AE and RT are the most common exercise therapies. Studies have shown that AE can significantly improve the four lipids in postmenopausal women [15], but RT can result only in significant improvements in TC and LDL-C in postmenopausal women [16]. Studies have also shown that CT is more effective in improving vascular function and lipids than AE or RT [17]. Compared to land-based exercise, WE has been shown to be more advantageous in improving PWV and BP in patients with peripheral arterial disease [18].

In summary, although numerous randomized controlled trials (RCTs) with pairwise meta-analyses have examined the efficacy of exercise interventions on vascular and lipid health in postmenopausal women, pairwise meta-analyses cannot rank the efficacy of different interventions, and it is not possible to determine which exercise intervention is better at improving vascular and lipid outcomes in postmenopausal women. Therefore, in this study, a network meta-analysis (NMA) of the effects of different exercise interventions on vascular function and lipids in postmenopausal women was conducted to investigate the optimal exercise modality. PWV, SBP, DBP, TC, TG, LDL-C, and HDL-C were used as outcome indicators to investigate the effects of exercise. A comprehensive assessment and ranking of the efficacy of the intervention on the outcome indicators was performed.

## 2. Materials and Methods

This systematic review and NMA are reported in accordance with the Preferred Reporting Items for Systematic Reviews and Meta-Analyses for Network Meta-Analyses (PRISMA-NMA) [19]. The study protocol was registered in the PROSPERO International Prospective Register of Systematic Reviews (Registration number: CRD42022336324).

### 2.1. Search Strategy

This study conducted a literature search in PubMed, EBSCO, Embase, The Cochrane Library, Web of Science, and other databases based on PICOS principles from the time of creation to March 2022, and tracked the references of the included literature. PICOS principles include: (1) P (participant): postmenopausal women, elderly women; (2) I (intervention): exercise; (3) C (comparison): exercise intervention or no-exercise control; and (4) O (outcome). The main search terms were postmenopausal women, exercise, training, cardiovascular, vascular function, TC, and TG. The detailed search strategy is shown in Supplementary Table S1.

### 2.2. Study Selection

The literature was screened with the aid of Endnote software, a literature check was performed to eliminate duplicates, two reviewers independently performed the reading of the title and abstract, and the screening was carried out according to the inclusion and exclusion criteria, with any disagreements resolved through discussion or discussion with a third party.

Inclusion criteria: (1) study type: RCTs; (2) study population: postmenopausal women, and more than 60 years of age; (3) interventions: AE, RT, CT, and WE (the detailed classifications of exercise training are shown in Table 1); (4) outcome indicators: PWV, SBP, DBP, TC, TG, LDL-C, and HDL-C; and (5) the studies must be published in English.

Exclusion criteria: (1) duplicate publications; (2) review papers and conference papers; (3) exercise combined with other therapies; (4) experimental animal studies; (5) full text or other relevant information not available after contacting the authors (n = 3).

### 2.3. Data Extraction

Two reviewers read, evaluated, and extracted data from the literature that met the inclusion criteria, reading the titles and abstracts to exclude any literature that was clearly ineligible. The extraction of the literature included (1) basic information about the included literature (title, author, and year of publication); (2) subject characteristics (sample size, age, and country); (3) exercise interventions (type of exercise, intensity, duration, frequency, and length of intervention); and (4) outcome indicators (PWV, SBP, DBP, TC, TG, LDL-C, and HDL-C).

### 2.4. Risk of Bias in Individual Studies

The quality of the included studies was assessed using the Cochrane system’s risk of bias (ROB) assessment criteria, with assessment form entries containing random sequence generation, allocation sequence concealment, blinding of subjects and researchers, blinding of outcome measures, incomplete outcome data, selective reporting, and other potential sources of bias [23].

### 2.5. Statistical Analysis

A meta-analysis of the included literature was performed using Stata 16.0 and Review Manager 5.3 software, with outcome indicators as continuous variables, using mean difference (MD) for the same unit of measurement and standardized mean difference (SMD) and 95% confidence (95% CI) as effect size indicators for different units of measurement. The heterogeneity (I^2^) and *p* values of the direct comparison between the different exercise modalities and the control group were first obtained by pairwise meta-analysis, followed by NMA, in which the size of the dots in the net evidence plot represents the sample size, and the line between the dots indicates the existence of a direct comparison between the two exercise modalities. The higher the number of direct comparison studies between the two interventions, the thicker the line, and the thinner the line otherwise [24]. If there was no line of connection between the two exercise modalities, indirect comparisons were made using NMA. Inconsistency tests were performed using nodal analysis, and if *p* > 0.05, a consistency model was used for analysis. Local inconsistency tests were also performed using nodal splits. When direct and indirect evidence were inconsistent, only a pairwise meta-analysis was performed. The ranking between individual interventions was obtained by comparing the surface under the cumulative ranking (SUCRA) in a cumulative probability plot, where 0 ≤ SUCRA ≤ 100, with 100 being the most effective for the treatment and 0 being the worst and ineffective, and with larger values denoting more effective interventions [25].

## 3. Results

### 3.1. Literature Selection

**Figure 1:**
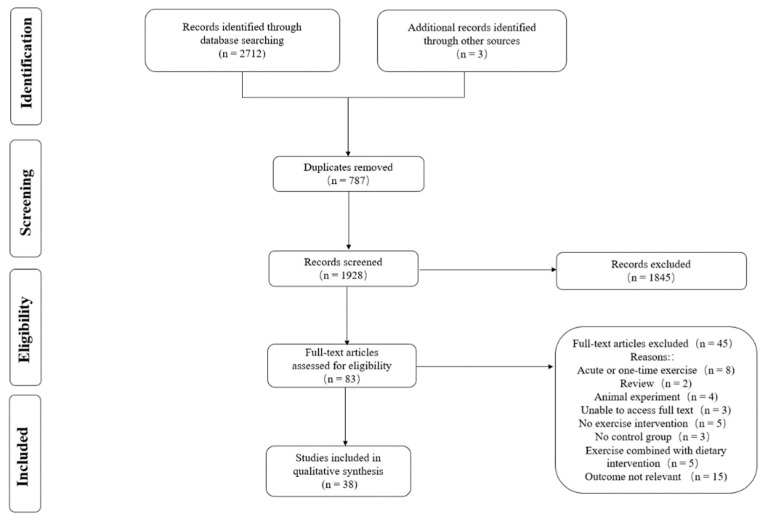
PRISMA flow diagram.

The search through various databases yielded 2712 papers, and three additional papers were obtained manually, resulting in a total of 2715 papers. After utilizing the literature management software EndNoteX9 to remove duplicates, a total of 1928 publications were included. After the initial screening by reading the titles and abstracts of the literature, 1845 irrelevant publications were excluded, leaving 83 publications. After further reading of the full texts, 45 publications were excluded, resulting in a total of 38 RCTs included in the NMA. The detailed process for the study search and selection is presented in Figure 1.

### 3.2. Characteristics of the Included Studies

A total of 1407 people were included in the 38 studies, with a total of 875 in the experimental group and 532 in the control group. The main interventions applied in the experimental group were AE (11), RT (13), CT (8), and WE (9), while the control group did not perform any exercise. The basic characteristics of the included studies (n = 38) are shown in Tables S2 and S3.

### 3.3. ROB

Of the 38 included studies, five reported the method of random allocation as a random number table method or random computer generation; one was coded according to physician judgment, and the remaining 32 did not describe the method of allocation in detail. Due to the nature of the intervention, only three of the included studies reported blinded methods, two double-blinded and one single-blinded, while the remaining studies did not describe them in detail. However, all studies met the blinding requirements for outcome indicators and had good data integrity to avoid selective reporting. The details of the ROB are shown in Figure S1.

### 3.4. Pairwise Meta-Analysis and NMA

#### 3.4.1. PWV

The pairwise meta-analysis results showed that exercise effectively improved PWV for the intervention (SMD = −1.53; 95% CI: −2.84, −0.22; *p* = 0.02; I^2^ = 95%; studies: n = 8; Figure S2), and high heterogeneity was found in the Wong 2018 study using an article-by-article exclusion approach. This literature resulted in significantly lower levels of heterogeneity (I2 = 37, *p* = 0.15) and a combined effect size (SMD = −0.57, 95% CI: −0.93, −0.21; *p* = 0.002). NMA could not be performed due to the small number of PWV indicators included in the study.

#### 3.4.2. SBP

**Figure 2:**
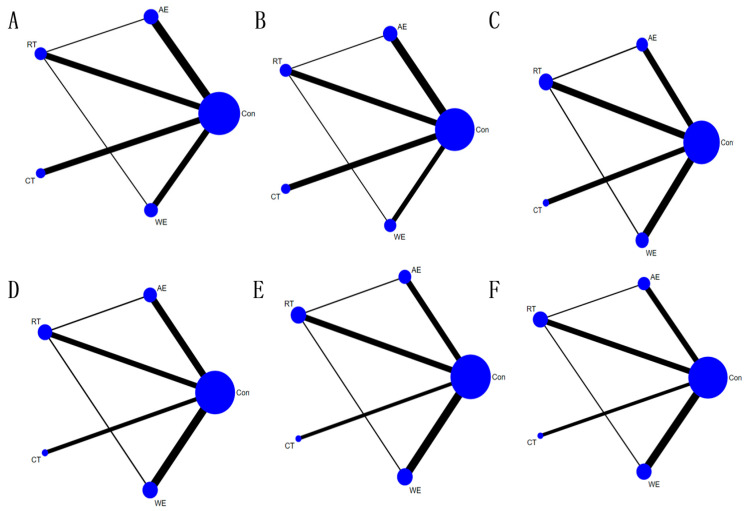
Evidence network diagram of network meta-analysis comparisons. Each node represents one exercise type. The lines between the dots indicate a direct comparison between the two modes of motion, with thicker lines for more studies and thinner lines for fewer studies. (**A**) Systolic blood pressure, (**B**) diastolic blood pressure, (**C**) total cholesterol, (**D**) triglycerides, (**E**) low-density lipoprotein cholesterol, (**F**) high-density lipoprotein cholesterol. AE, aerobic exercise; RT, resistance training; CT, combined training; WE, water exercise; CON, control group.

**Figure 3:**
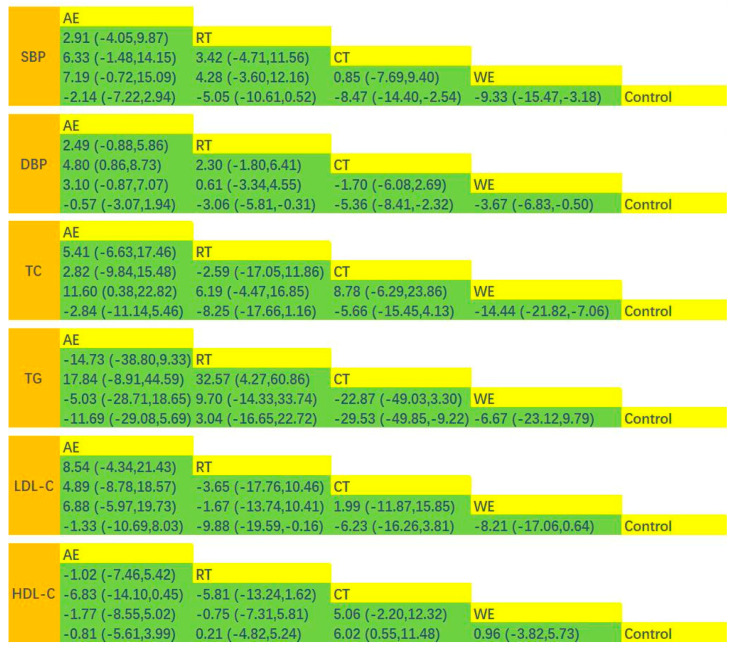
Matrix of the network meta-analysis results. AE, aerobic exercise; RT, resistance training; CT, combined training; WE, water exercise; CON, control group. Each cell shows the MD, along with the 95% CI.

The results of pairwise meta-analysis showed that exercise effectively improved SBP in the intervention group (MD = −5.65; 95% CI: −7.98, −3.33; *p* < 0.00001; I^2^ = 73%; study n = 23; Figure S3). NMA-based concordance analysis showed that CT (MD = −8.47, 95% CI: −14.40, −2.54, *p* < 0.05) and WE (MD = −9.33, 95% CI: −15.47, −3.18, *p* < 0.05) were significantly more effective than the control in reducing SBP. AE (MD = −0.81, 95% CI: −5.61, 3.99, *p* > 0.05) and RT (MD = 0.21, 95% CI: −4.82, 5.24, *p* > 0.05) showed no statistically significant difference in results compared to the control group. Indirect comparisons between the two showed no statistically significant differences between the two intercomparisons for each exercise modality (*p* > 0.05; Figure 2A and Figure 3). The order of superiority of the different exercise modalities in improving SBP was WE (SUCRA = 84.9), CT (SUCRA = 79.1), RT (SUCRA = 52.4), AE (SUCRA = 27.3), and control group (SUCRA = 6.3) (Figure S9).

#### 3.4.3. DBP

The results of pairwise meta-analysis showed that exercise effectively improved DBP in the intervention group (MD = −2.85; 95% CI: −3.27, −2.43; *p* < 0.00001; I^2^ = 32%; study n = 22; Figure S4). NMA-based concordance analysis showed that RT (MD = −3.06, 95% CI: −5.81, −0.31, *p* < 0.05), CT (MD = −5.36, 95% CI: −8.41, −2.32, *p* < 0.05), and WE (MD = −3.67, 95% CI: −6.83, −0.50, *p* < 0.05) were significantly more effective than the control in reducing DBP. Regarding AE (MD = −0.57, 95% CI: −3.07, 1.94, *p* > 0.05), the results were not statistically different compared to the control group. An indirect comparison between the two showed that CT was more effective than AE in two areas (MD = −4.80, 95% CI: −8.73, −0.86), while the other differences between the two intercomparisons were not statistically significant (*p* > 0.05; Figure 2B and Figure 3). The order of superiority of the different exercise modalities in improving DBP was CT (SUCRA = 91.1), WE (SUCRA = 69.1), RT (SUCRA = 60.5), AE (SUCRA = 20.3), and control group (SUCRA = 9.1) (Figure S10).

#### 3.4.4. TC

The results of the pairwise meta-analysis showed that exercise effectively improved TC in the intervention group (MD = −5.23; 95% CI: −9.00, −1.45; *p* = 0.007; I^2^ = 0%; study n = 18; Figure S5). NMA-based concordance analysis showed that WE (MD = −14.44, 95% CI: −21.82, −7.06, *p* < 0.05) was significantly more effective than the control in reducing TC. AE (MD = −2.84, 95% CI: −11.14, 5.46, *p* > 0.05), RT (MD = −8.25. 95% CI: −17.66, 1.16, *p* > 0.05), and CT (MD = −5.66, 95% CI: −15.45, 4.13, *p* > 0.05) showed no statistically significant difference in results compared to the controls. Indirect comparisons between the two showed no statistically significant differences between the two intercomparisons for each exercise modality (*p* > 0.05; Figure 2C and Figure 3). The order of superiority of the different exercise modalities in improving TC was WE (SUCRA = 93.1), RT (SUCRA = 63.2), CT (SUCRA = 50.8), AE (SUCRA = 31.1), and control group (SUCRA = 10.7) (Figure S11).

#### 3.4.5. TG

The results of the pairwise meta-analysis showed that exercise effectively improved TG in the intervention group (MD = −12.46; 95% CI: −20.97, −3.95; *p* = 0.004; I^2^ = 92%; study n = 18; Figure S6). NMA-based concordance analysis showed that CT (MD = −29.53, 95% CI: −49.85, −9.22, *p* < 0.05) was significantly more effective than the control in reducing TG. AE (MD = −11.69, 95% CI: −29.08, 30.98, *p* > 0.05), RT (MD = 3.04. 95% CI: −16.65, 22.72, *p* > 0.05), and WE (MD = −6.67, 95% CI: −23.12, 9.79, *p* > 0.05) showed no statistically significant difference in the results compared to the controls. Indirect comparison between the two showed that CT was more effective than RT (MD = −17.84, 95% CI: −44.59, 8.91), while the other differences between the two intercomparisons were not statistically significant (*p* > 0.05; Figure 2D and Figure 3). The order of superiority of the different exercise modalities in improving TG was CT (SUCRA = 96.2), AE (SUCRA = 63.7, WE (SUCRA = 48.9), RT (SUCRA = 17.9), and control group (SUCRA = 23.3) (Figure S12).

#### 3.4.6. LDL-C

The results of the pairwise meta-analysis showed that exercise effectively improved LDL-C in the intervention group (MD = −4.42; 95% CI: −7.86, −0.97; *p* = 0.01; I^2^ = 31%; study n = 18; Figure S7). NMA-based concordance analysis showed that RT (MD = −9.88, 95% CI: −19.59, −0.16, *p* < 0.05) was significantly more effective than the control in reducing LDL-C. AE (MD = −1.33, 95% CI: −10.69, 8.03, *p* > 0.05), CT (MD = −6.23, 95% CI: −16.26, 3.81, *p* > 0.05), and WE (MD = −8.21, 95% CI: −17.06, 0.64, *p* > 0.05) showed no statistically significant difference in results compared to the control group. Indirect comparisons between the two showed no statistically significant differences between the two intercomparisons for each exercise modality (*p* > 0.05; Figure 2E and Figure 3). The order of superiority of the different exercise modalities in improving LDL-C was RT (SUCRA = 79.4), WE (SUCRA = 70.3), CT (SUCRA = 58.9), AE (SUCRA = 27.3), and control group (SUCRA = 14.1) (Figure S13).

#### 3.4.7. HDL-C

The results of the pairwise meta-analysis showed that the intervention group was not statistically significant compared to the control group (MD = −1.44; 95% CI: −1.30, 4.18; *p* = 0.30; I^2^ = 86%; study n = 19; Figure S8). NMA-based concordance analysis showed that CT (MD = 6.02, 95% CI: 0.55, 11.48, *p* < 0.05) was significantly more effective than the control in improving HDL-C. AE (MD = −0.81, 95% CI: −5.61, 3.99, *p* > 0.05), RT (MD = 0.21, 95% CI: −4.82, 5.24, *p* > 0.05), and WE (MD = −0.96, 95% CI: −3.82, 5.73, *p* > 0.05) showed no statistically significant difference in results compared to controls. Indirect comparisons between the two showed no statistically significant differences between the two intercomparisons for each exercise modality (*p* > 0.05; Figure 2F and Figure 3). The order of superiority of the different exercise modalities in improving HDL-C was CT (SUCRA = 94.8), WE (SUCRA = 50.8), RT (SUCRA = 41.0), AE (SUCRA = 27.2), and control group (SUCRA = 36.2) (Figure S14).

### 3.5. Publication Bias or Small Sample Effect Test

The indexes involved in the study were tested for publication bias (Figures S15–S20). The indexes for SBP and LDL-C were asymmetric in the funnel plots, suggesting that there was a certain publication bias or small sample effect, which may have had a certain impact on the results of the corresponding indexes. The funnel plots for the other indicators were basically symmetrical, suggesting that there was a low possibility of publication bias or a small sample effect in the current study.

## 4. Discussion

This study compared the effects of different exercise interventions on vascular function and blood lipid levels in postmenopausal women. A total of 38 RCTs with four exercise interventions were included, with a total sample size of 1407 participants. The results of the study showed that exercise was effective in improving PWV, SBP, and DBP, and TC, TG, and HDL-C levels, in postmenopausal women. Furthermore, WE was the most effective intervention in improving TC and SBP; CT was the most effective in improving TG, HDL-C, and DBP; and RT achieved good results in improving LDL-C.

### 4.1. Effect of Exercise on PWV, SBP, and DBP in Postmenopausal Women

PWV has been widely used in clinical practice as a method for evaluating AS. Studies have noted that PWV levels have significant predictive value for cardiovascular mortality and morbidity [26]. Regular exercise can prevent and delay the development of atherosclerotic plaques and reduce the risk of CVD in postmenopausal women [27]. Studies have shown that exercise can improve arterial stiffness in postmenopausal women in different health states [28]. The results of the pairwise meta-analysis in this study showed that exercise significantly improved PWV in postmenopausal women. Due to the small number of studies included in the PWV index, an NMA could not be performed. The improvement mechanism of exercise on arterial stiffness in postmenopausal women may be that repeated hemodynamic stimulation in each exercise acts on the vascular wall, resulting in a change in arterial diameter and structural remodeling [13]. In addition, reduced oxidative stress after exercise is also beneficial to regulate the number and function of endothelial progenitor cells to promote endothelial homeostasis [29], increase shear force and mitochondrial biosynthesis, induce an anti-inflammatory response, and inhibit tumor necrosis factor-α (TNF-α). It has a protective effect on TNF-α-induced vascular injury [30].

In terms of SBP and DBP, a meta-study demonstrated the significance of SBP reduction for cardiovascular health, in that every 10 mmHg reduction in SBP would reduce the risk of cardiovascular disease by 20% and total mortality by 13% [31]. There was a J-curve relationship between DBP levels and adverse cardiovascular events, with low DBP levels associated with an increased risk of all-cause mortality and myocardial infarction [32,33]. Kazeminia et al. found that regular exercise was beneficial in improving BP abnormalities [34]. The results of this study showed that exercise can significantly improve SBP and DBP levels in postmenopausal women, and the NMA found that WE was the best exercise to improve SBP levels, and CT was the best in improving DBP. The meta-analysis by Zhou et al. found that WE was effective in improving SBP levels in postmenopausal women [35], which is consistent with the results of this study. The mechanism of improvement of arterial stiffness in postmenopausal women by WE is mainly through the following aspects. (1) As the body can accelerate blood circulation by exercising in water, it can increase the impact and shear stress of blood on the inner wall of blood vessels (shear stress), take away the deposits on the inner wall of blood vessels, reduce peripheral resistance, and improve the compliance of peripheral blood flow, resulting in lower SBP [36]. (2) WE can also reduce SBP by increasing blood NO, improving vascular endothelial function, and helping vasodilation [37]. (3) The effect of hydrostatic pressure on pressure receptor stimulation promotes venous return and stimulates pressure receptors to trigger an increase in cardiac filling and output per beat, reflexively lowering the heart rate and blood pressure [38]. Cornelissen et al. showed that combined exercise significantly reduced DBP [39]. The European Association of Preventive Cardiology (EAPC) suggested, in the European Consensus on the Prescription of Exercise to Lower BP, that CT could be an effective alternative to non-pharmacological treatment in patients with hypertension [40]. The mechanism associated with CT to improve DBP levels in postmenopausal women is mainly through the complementary effects of AE and RT. AE significantly reduces BP in postmenopausal hypertensive women by improving endothelial function, nitric oxide levels, and arterial compliance, and reducing insulin resistance [41,42,43], whereas postmenopausal women typically experience a decrease in muscle strength and mass, which is associated with increased arterial stiffness [44], which can lead to abnormal BP levels [45]. Therefore, CT, which combines AE and RT to increase muscle strength and mass in postmenopausal women through RT, can better improve BP levels and maximize the benefits of AE.

### 4.2. Effect of Exercise on Blood Lipid Levels in Postmenopausal Women

Current studies have confirmed a close relationship between TC and AS and coronary heart disease [46,47,48]. The present study showed that exercise significantly improved TC indicators in postmenopausal women for whom NMA was performed and found that WE was the best exercise for improving TC levels. Currently, studies have verified that WE can significantly improve TC levels in dyslipidemic populations [49]. The mechanism of improvement in TC levels in postmenopausal women by WE may be related to the following: the improvement in TC levels may be related to the higher baseline TC levels in the subjects included in this study, which were higher than 200 mg/dL in 15 of the included studies, and dyslipidemia is usually diagnosed when TC exceeds 200 mg/dL; during exercise, the body’s consumption of energy substances and excretion of metabolites are higher than when it is quiet. Due to the special characteristics of the water environment, the resistance to exercise in water is greater than that of exercise on land, and the energy expended when conducting the same speed of movement in water is approximately six times higher than that on land. Therefore, WE can be more effective in improving TC levels in postmenopausal women. The significant improvement in the TC index may also be related to the reduction in LDL-C. LDL-C occupies a high proportion of TC, and this study showed that exercise can significantly improve LDL-C in postmenopausal women. Therefore, the decrease in LDL-C may be another reason for the decrease in TC. In this study, NMA on LDL-C found that RT was the best exercise to improve LDL-C levels. It was shown that the improvement in LDL-C levels through exercise may be related to the improved activity of low-density lipoprotein receptor (LDL-R) in the hepatocyte membrane, which reduces LDL-C levels in the body by mediating and inducing LDL-C into the hepatocyte for metabolism. Meanwhile, abnormal LDL-R is a cause of hypercholesterolemia, and exercise promotes LDL-R activity and expression on cell membranes, improving LDL-C levels [50]. Another possibility is that the expression of LDL receptor messenger ribonucleic acid (LDL-R mRNA) is enhanced due to increased insulin sensitivity, accelerating the rate of LDL-C metabolism [51]. However, the exact mechanism by which RT intervenes in this pathway is not fully understood, but studies have demonstrated the benefits of RT on LDL-C. Cunha et al. found that RT improved LDL-C levels in middle-aged and older adults, but had no effect on TG or HDL-C levels [52].

Most studies have shown that HDL-C is a prognostic marker of CVD [53], and a slight improvement in its concentration is significantly associated with CVD [54]. Mann et al. found that CT has the best effect on reducing blood lipid levels, such as HDL-C [55]. The results of this study showed that exercise had no statistical significance in improving HDL-C in postmenopausal women. An NMA found that CT was the best exercise to improve HDL-C in postmenopausal women. This result may be related to the subjects’ diet, and studies by Garg [56] found that a high-carbohydrate diet strategy can significantly reduce blood HDL-C levels. He et al. studied a 12-week exercise and diet control intervention in postmenopausal women and found a beneficial HDL increase only in the combination group of exercise and diet control (reduced 500–1000 kcal calories per day), and the single exercise intervention group and the single diet control group showed no improvement [57]. However, none of the included studies in this review conducted dietary interventions on the subjects. Therefore, it may be that there is no improvement in the HDL-C index due to the lack of intervention in the subjects’ diets. TG is the most abundant lipid in the human body [58], and excessive TG is a risk factor for CVD [59]. The results of this study showed that exercise can significantly improve TG levels in postmenopausal women, and an NMA found that CT was the best exercise to improve TC levels. The improvement in TG by exercise may be related to the levels of adipocytokines, including leptin, adiponectin (ADPN), TNF-α, plasminogen activator inhibitor-1 (PAI-1), and interleukin-6. Studies have confirmed that abnormal adipocytokines are one of the factors that cause blood lipid problems, and exercise can reduce leptin and increase ADPN [60]. The improvement in TG may also be related to hormone-sensitive lipase (HSL). Exercise promotes the activity of HSL in muscle cells, accelerates the decomposition of TG into free fatty acids, enters the mitochondria for oxidation and energy supply, and helps to reduce the concentration of TG [61,62]. Tambalis et al. found that CT has the best effect on reducing blood lipid levels such as TG and HDL-C [63]. The improvement mechanism of CT on TG and HDL-C in postmenopausal women can be attributed to the cross-influence of AE combined with RT on the body [64,65,66]. Compared with a single exercise method, the combination of AE and RT can reduce body fat content more effectively [67], and excessive fat content is the main reason for abnormal lipid metabolism.

## 5. Strengths and Limitations

This study used NMA to examine the effects of different exercise modalities on vascular function and blood lipids in postmenopausal women, which can reflect the benefits of different exercise modalities on vascular function and blood lipids in postmenopausal women and provide reference information for choosing the appropriate exercise modality. However, there are several limitations to this study. (1) Although this study verified the benefits of various exercise modalities on vascular function and blood lipids in postmenopausal women, the number of included papers was limited, and it was not possible to classify the analysis according to the intensity and duration of exercise. (2) The number of original studies included in the PWV index was small, and no NMA could be conducted. Future studies should focus on this index to further improve the reliability and persuasiveness of the study. (3) The number of original studies that included WE was small, and the analysis could not be carried out according to the type of exercise, water temperature, or water depth.

## 6. Conclusions

Exercise can effectively improve the PWV, SBP, and DBP and the levels of TC, TG, and LDL-C in postmenopausal women. An NMA showed that WE has the highest probability of becoming the optimal exercise mode in reducing TC and SBP. CT had the best effect on improving TG, HDL-C, and DBP. To improve LDL-C, RT can achieve a good effect. However, considering the limitations of the above mesh meta-analysis, the results should be interpreted with caution. Furthermore, more RCTs should be conducted in the future to provide more direct evidence of the efficacy of various exercise interventions on vascular health in postmenopausal women.

## Figures and Tables

**Table 1:** Classifications of exercise training.

Type	Definition
Aerobic exercise (AE)	Exercise training designed to improve the efficiency and capacity of the cardiorespiratory system [20].
Resistance training (RT)	Exercise training designed to improve the strength, power, endurance, and size of skeletal muscles [21].
Combined training (CT)	A combination of AE and RT.
Water exercise (WE)	Refers to the use of physical and other characteristics of water for exercise in the water environment, including AE in water and RT in water [22].

## Data Availability

The original contributions presented in the study are included in the article/Supplementary Material. Further inquiries can be directed to the corresponding author.

## Supplementary Materials

The following supporting information can be downloaded at: https://www.mdpi.com/article/10.3390/ijerph191912074/s1, Table S1: Search strategy for Pubmed; Table S2: Basic Information of the Included Studies; Table S3: Exercise Intervention and Outcome Index of the Included Studies; Figure S1: Cochrane risk bias evaluation chart; Figure S2: Pairwise Meta-analysis of exercise on PWV; Figure S3: Pairwise Meta-analysis of exercise on SBP; Figure S4: Pairwise Meta-analysis of exercise on DBP; Figure S5: Pairwise Meta-analysis of exercise on TC; Figure S6: Pairwise Meta-analysis of exercise on TG; Figure S7: Pairwise Meta-analysis of exercise on LDL-C; Figure S8: Pairwise Meta-analysis of exercise on HDL-C; Figure S9: Cumulative ranking probability plots for SBP; Figure S10: Cumulative ranking probability plots for DBP; Figure S11: Cumulative ranking probability plots for TC; Figure S12: Cumulative ranking probability plots for TG; Figure S13: Cumulative ranking probability plots for LDL-C; Figure S14: Cumulative ranking probability plots for HDL-C; Figure S15: Network meta-analysis funnel plots for SBP; Figure S16: Network meta-analysis funnel plots for DBP; Figure S17: Network meta-analysis funnel plots for TC; Figure S18: Network meta-analysis funnel plots for TG; Figure S19: Network meta-analysis funnel plots for LDL-C; Figure S20: Network meta-analysis funnel plots for HDL-C.
