# Supplementary material for: Effect of Exercise on Vascular Function and Blood Lipids in Postmenopausal Women: A Systematic Review and Network Meta-Analysis
Source: Int J Environ Res Public Health. 2022 Sep 23;19(19):12074. doi: 10.3390/ijerph191912074 (PMC9564891; doi:10.3390/ijerph191912074)

Supplementary Material

Content	Pages
Table S1. Search strategy.	2
Table S2. Basic Information of the Included Studies	3-4
Table S3. Exercise Intervention and Outcome Index of the Included Studies	5-7
Figure S1. Cochrane risk bias evaluation chart.	8
Figure S2-8. Pairwise Meta-Analysis.	9-11
Figure S9-14. Ranking of exercise interventions in order of effectiveness.	12-13
Figure S15-20. Publication Bias or Small Sample Effect Test	14-15

Table S1 Search strategy for Pubmed

	Search strategy
**Pubmed**	((((((((Menopausal women[Title/Abstract]) OR (postmenopausal women[Title/Abstract])) OR (postmenopausal female[Title/Abstract])) OR (menopause female[Title/Abstract])) OR (elderly women[Title/Abstract])) OR (older adults[Title/Abstract])) OR (postmenopausal females[Title/Abstract])) AND (((((((cardiovascular[Title/Abstract]) OR (flow-mediated dilation[Title/Abstract])) OR (pulse wave velocity[Title/Abstract])) OR (intima media thickness[Title/Abstract])) OR (vascular function[Title/Abstract])) OR (total cholesterol[Title/Abstract])) OR (triglyceride[Title/Abstract]))) AND (((((((Exercise[Title/Abstract]) OR (physical activit*[Title/Abstract])) OR (training*[Title/Abstract])) OR (resistance training[Title/Abstract])) OR (aerobic exercise[Title/Abstract])) OR (combined training[Title/Abstract])) OR (water exercise[Title/Abstract]))) AND (((randomized controlled trial[Title/Abstract]) OR (randomized[Title/Abstract])) OR (placebo[Title/Abstract]))

Table S2 Basic Information of the Included Studies

Study	Country	Group	Age（ Mean ± SD）	n
James1991	USA	AE	47 ±2 years	11
		RT	47 ±2 years	12
Casey2007	USA	AE	59.7±6.5 years	10
		RT	58.7±4.5 years	13
Zois2009	Greece	CT	55.0 ±5.2 years	10
		CON	59.4±3.2 years	10
Arturo2011	USA	CT	54 ±2years	12
		CON	54 ±1 years	12
Masanori2012	Japan	AE	72 ±4.2 years	13
		CON	71.5±7.4 years	13
Nihal2012	Turkey	RT	54.33 ± 5.3 years	24
		CON	51.80 ± 3.65 years	21
Asako2012	Japan	AE	60±6 years	11
		CON	60±7 years	11
Katie2012	USA	CT1	65.6±0.7 years	27
		CT2	63.7±0.5 years	30
		CT3	64.8±0.7 years	22
Figueroa2012	USA	RT	56±3 years	15
		CON	56±3 years	13
Gerage2013	Brazil	RT	65.5 ± 5.0 years	15
		CON	66.2 ± 4.1 years	14
Cleiton2014	Brazil	RT1	59.5 ± 6.3years	12
		RT2		11
		CON		12
Dos2014	Brazil	CT1	64.2 ± 3.1 years	20
		CT2	62.6 ± 2.5 years	20
		CON	63.1 ± 2.3 years	20
Camilla2016	Danmark	AE	53.4 ± 3 years	38
Nishida2015	Japan	AE	70.4± 5.8 years	31
		CON	69.7 ± 6.6 years	31
Wong2016	USA	RT	59 ±1 years	13
		CON	58±1 years	12
Azadpour2017	Turkey	AE	57.58 ±4.29 years	12
		CON	56.58 ±4.17 years	12
Jinkee2017	Japan	CT	73.5±7.1 years	25
		CON	74.7±5.1 years	25
Won2017	Korea	CT	76±5 years	10
		CON	74.7±2 years	10
Anahita2018	Iran	CT	54.83±4.72 years	12
		CON	56.9±4.93 years	12

Alexei2018	Korea	RT	59±1 years	20
		CON	59±1 years	21
Wong2018	Korea	WE	74±4 years	52
		CON	73±4 years	48
Tze2019	Australia	AE	53.9±3.39 years	30
		CON	53.2±3.07 years	30
Eun2019	Korea	AE1	61.8±10.1 years	21
		AE2	57.3±8.4 years	13
		CON	62.5±13.9 years	13
Paolo2019	Brazil	RT	71.40 ± 5.71 years	25
		CON	69.04 ± 4.45 years	23
Leandro2020	Brazil	RT1	67.3 ± 4.4 years	20
		RT2		20
		CON		19
Won2020	Korea	RT	67.4 ± 1.1 years	10
		CON	67.7 ± 1.0 years	10
HuiHe2022	China	AE	58.3 ± 3.0 years	15
		CON	57.6 ± 3.2 years	14
Arca2014	Brazil	WE	64 ± 7.0 years	19
		CON		14
Colado2009	Spain	WE	54.7±2.0 years	15
		RT	54.0±2.8 years	21
		CON	52.9±1.9 years	10
Woo2016	Korea	WE	73.22±3.30 years	25
		CON	72.65±4.28 years	15
Sattar2012	Iran	WE	55.2 ± 3.7 years	14
		CON	54.4 ± 4.6 years	10
Pak2014	China	WE	69.4 ± 7.19 years	13
		CON	74.6 ± 6.52 years	10
Rodrigo2008	Australia	WE	53.1±1.9 years	36
		CON	54.3±2.5 years	22
Takeshima2002	Japan	WE	69.3±4.5 years	15
		CON	69.3±3.3 years	15
Kim2018	Korea	WE	66.77±3.07 years	14
		AE	67.42±1.78 years	14
		CON	66.43±4.45 years	12
Kim2021	Korea	WE	74.36 ± 3.78 years	12
		CON	75.90 ± 4.23 years	10
Arturo2013	USA	RT	54 ±1 years	14
Jeon2018	Korea	CT	59 ±1 years	8
		CON	58 ±2 years	8

Abbreviations: AE, aerobic exercise; RT, resistance training; CT, combined training; WE, water exercise; CON, control group.

Table S3 Exercise Intervention and Outcome Index of the Included Studies

Study	Group	Intervening measure and intensity	Intervention duration (minutes per session, times per week, total weeks)	Outcome Index
James1991	AE<br>RT	running；70%HRR<br>65-70% 1RM	50 min, 3d/w, 12w<br>55 min, 3d/w, 12w	TC、TG、LDL-C、HDL-C
Casey2007	AE<br>RT	running；65-80%HRR<br>65-70% 1RM	40 min, 2d/w, 18w<br>35 min, 2d/w, 18w	SBP、DBP
Zois2009	CT<br>CON	60% 1RM for RT+70% HRmax for AE<br>No exercise	75 min, 2d/w, 16w	TC、TG、LDL-C、HDL-C
Arturo2011	CT<br>CON	60% 1RM for RT+60% HRmax for AE<br>No exercise	40 min, 3d/w, 12w	SBP、DBP、PWV
Masanori2012	AE<br>CON	15RPE<br>No exercise	20min, 7d/w, 12w	TC、TG、LDL-C、HDL-C、SBP、DBP、PWV
Nihal2012	RT<br>CON	60% 1RM<br>No exercise	50min, 3d/w, 12w	TC、TG、LDL-C、HDL-C、SBP、DBP、
Asako2012	AE<br>CON	walking/cycling；70%HRR<br>No exercise	45min, 3d/w, 8w	TC、TG、LDL-C、HDL-C、SBP、DBP、
Katie2012	CT1<br>CT2<br>CT3	80% 1RM for RT+70% HRmax for AE	45min, 1d/w, 16w<br>45min, 2d/w, 16w<br>45min, 3d/w, 16w	SBP、DBP
Figueroa2012	RT<br>CON	80% 1RM<br>No exercise	3d/w, 6w	SBP、DBP
Gerage2013	RT<br>CON	65% 1RM	30min, 3d/w, 12w	SBP、DBP
Cleiton2014	RT1<br>RT2<br>CON	65% 1RM<br>No exercise	20min, 3d/w, 12w<br>40min, 3d/w, 12w	TC、LDL-C、HDL-C
Dos2014	CT<br>CON	75% 1RM for RT+65-75% HRmax for AE<br>No exercise	50min, 3d/w, 16w	SBP、DBP
Camilla2016	AE	cycling	60min, 3d/w, 12w	TC、TG、LDL-C、HDL-C、
Nishida2015	AE<br>CON	stair stepping<br>No exercise	20min, 7d/w, 12w	TC、TG、LDL-C、HDL-C、SBP、DBP、
Wong2016	RT<br>CON	whole body vibration training<br>No exercise	60min, 3d/w, 8w	SBP、DBP
Azadpour2017	AE<br>CON	running；1-2 weeks, 50% HRmax ; 3-4 weeks, 55% HRmax；5-6 weeks, 60% HRmax；7-10weeks, 60% HRmax；<br>No exercise	40min, 3d/w, 10w	SBP、DBP
Jinkee2017	CT<br>CON	walking＋elastic bands；13-17RPE<br>No exercise	80min,5d/w, 24w	TC、TG、LDL-C、HDL-C、SBP、DBP
Won2017	CT<br>CON	running＋RT；1-4 weeks, 50% HRR ; 5-8weeks, 60% HRR；9-12 weeks, 70% HRR；<br>No exercise	70min,3d/w, 12w	SBP、DBP、PWV

Anahita2018	CT<br>CON	50-75% 1RM for RT+50-75% HRmax for AE<br>No exercise	90min,3d/w, 8w	TC、TG、LDL-C、HDL-C、SBP、DBP
Alexei2018	RT<br>CON	13RPE<br>No exercise	2-4d/w,12w	SBP、DBP、PWV
Wong2018	WE<br>CON	swimming；1-5 weeks, 60% HRmax ;6-20 weeks, 70-75% HRmax；<br>No exercise	30min,3-4d/w, 20w	SBP、DBP、PWV
Tze2019	AE<br>CON	cycling；13-14 RPE<br>No exercise	30min,3d/w, 8w	TC、TG、LDL-C、HDL-C、SBP、DBP、PWV
Eun2019	AE1<br>AE2<br>CON	running；40-80% HRmax<br>running/walking；60-80% HRmax<br>No exercise	50min,7d/w,12w	SBP、DBP
Paolo2019	RT<br>CON	65-75% 1RM<br>No exercise	20min,3d/w,12w	TC、TG、LDL-C、HDL-C、
Leandro2020	RT1<br>RT2<br>CON	65-80% 1RM<br>60-85%1RM<br>No exercise	3d/w,8w	TC、TG、LDL-C、HDL-C、
Won2020	RT<br>CON	elastic bands；1-4 weeks, 40-50% 1RM ;5-8 weeks, 50-60%1RM；9-12 weeks, 60-70%1RM<br>No exercise	60min,3d/w,12w	SBP、DBP
HuiHe2022	AE<br>CON	running；40%HRR<br>No exercise	50min,5d/w,12w	TC、TG、LDL-C、HDL-C、SBP、DBP
Arca2014	WE<br>CON	walking；40-50%HRR<br>No exercise	50min,3d/w,12w	TC、TG、LDL-C、HDL-C、SBP、DBP
Colado2009	WE<br>RT<br>CON	RT<br>elastic bands<br>No exercise	35-60min,2-3d/w,24w	TC、TG、LDL-C、HDL-C、SBP、DBP
Woo2016	WE<br>CON	60-80% HRmax<br>No exercise	50min,3d/w,12w	TC、TG、LDL-C、HDL-C、SBP、DBP
Sattar2012	WE<br>CON	RT<br>No exercise	45-60min, 3d/w,8w	SBP
Pak2014	WE<br>CON	Taichi<br>No exercise	60min,3d/w,8w	TG、LDL-C、HDL-C、 SBP、DBP
Rodrigo2008	WE<br>CON	RT<br>No exercise	2d/w,12w	TC、TG、LDL-C、HDL-C
Takeshima2002	WE<br>CON	walking +RT；60-80% HRmax<br>No exercise	70min,3d/w,12w	TC、TG、LDL-C、HDL-C、
Kim2018	WE<br>AE<br>CON	1-4weeks 40%-50%HRR;5-8weeks 50%-60% HRR; 9-12weeks 60%-65% HRR;13-16weeks 65%-70% HRR<br>No exercise	60min,2d/w,16w	PWV
Kim2021	WE<br>CON	1-4weeks 9-10RPE;5-8weeks 11-12RPE; 9-12weeks 13-14RPE<br>No exercise	60min,3d/w,12w	TC、LDL-C、HDL-C
Arturo2013	RT	60% 1RM	3d/w,12w	SBP、DBP
Jeon2018	CT<br>CON	walking＋RT；60- 80% HRmax<br>No exercise	60min,3d/w,12w	SBP 、DBP、TC、TG、PWV

Abbreviations: AE, aerobic exercise; RT, resistance training; CT, combined training; WE, water exercise; CON, control group; HRR, heart rate reserve; HRmax, maximal heart rate; RM, repetition maximum；RPE, Rating of perceived exertion；TC, total cholesterol；TG, triglyceride；LDL-C ,low density lipoprotein cholesterol；HDL-C, high density lipoprotein cholesterol; SBP, systolic blood pressure；DBP, diastolic blood pressure；PWV, pulse wave velocity.

Figure S1 Cochrane risk bias evaluation chart.

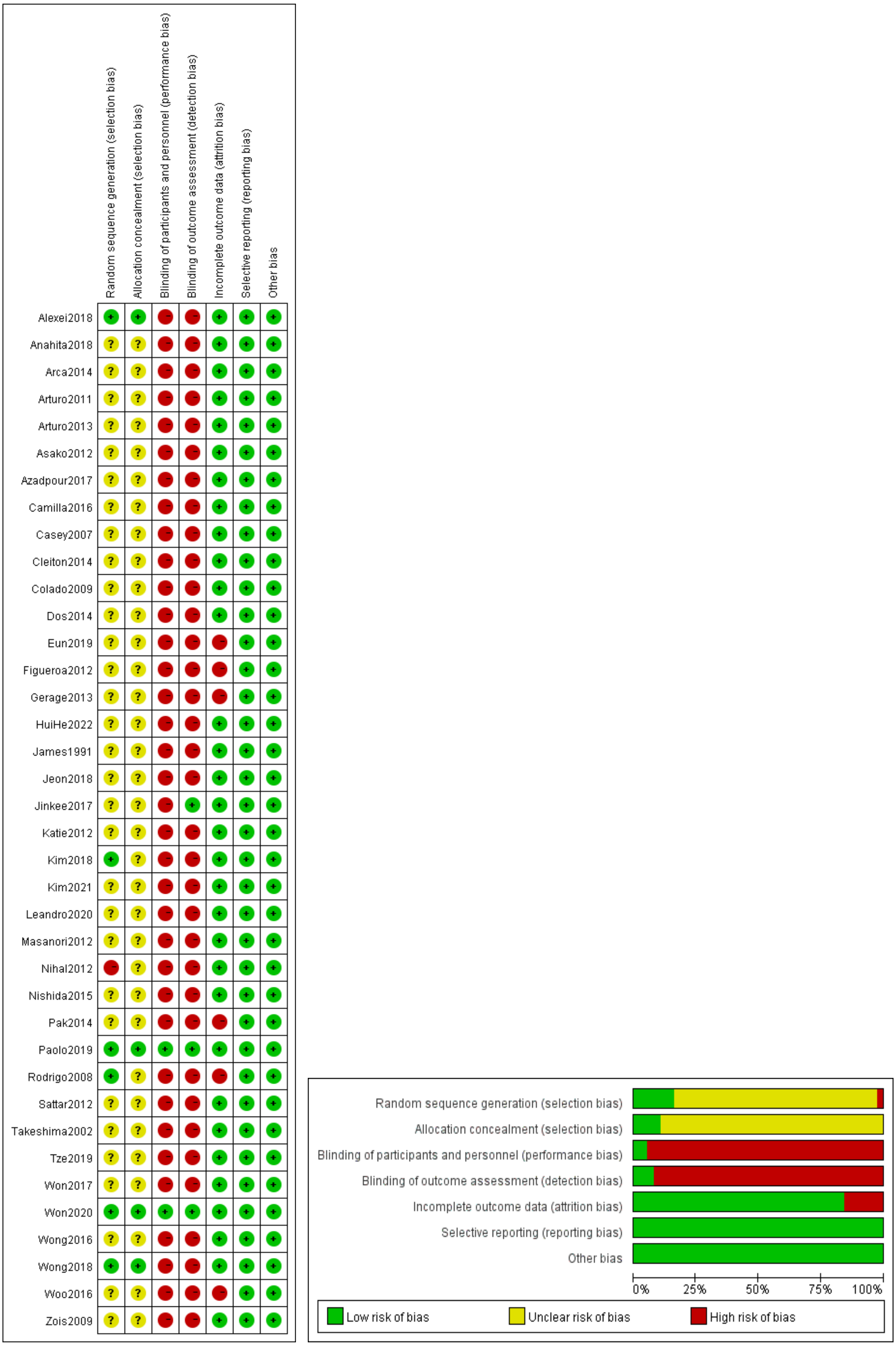

Figure S2 Pairwise Meta-analysis of exercise on PWV

Study or Subgroup	Experimental Mean	Experimental SD	Experimental Total	Control Mean	Control SD	Control Total	Weight	Std. Mean Difference IV, Random, 95% CI
Alexei2018	13.4	0.89	20	14.3	0.46	21	12.8%	-1.26 [-1.93, -0.58]
Arturo2011	12.14	2.04	12	12.73	1.56	12	12.6%	-0.31 [-1.12, 0.49]
Jeon2018	14.9	1.41	8	17.2	3.39	8	12.2%	-0.84 [-1.87, 0.20]
Kim2018	8.45	0.31	14	8.66	0.29	12	12.6%	-0.68 [-1.47, 0.12]
Masanori2012	16.58	1.65	13	18.47	2.54	13	12.6%	-0.85 [-1.66, -0.05]
Tze2019	7.12	1.37	30	7.22	1.27	30	13.0%	-0.07 [-0.58, 0.43]
Won2017	8.89	1.13	10	9.41	3.48	10	12.5%	-0.19 [-1.07, 0.69]
Wong2018	7.8	0.3	52	10	0.2	48	11.8%	-8.50 [-9.76, -7.23]
**Total (95% CI)**			**159**			**154**	**100.0%**	**-1.53 [-2.84, -0.22]**

Heterogeneity: Tau² = 3.39; Chi² = 154.90, df = 7 (P < 0.00001); I² = 95%
Test for overall effect: Z = 2.29 (P = 0.02)

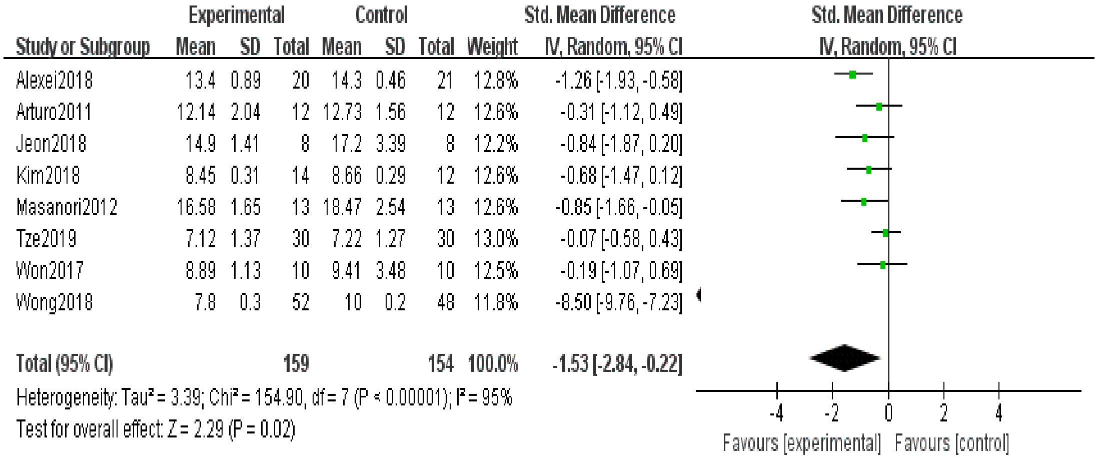

Figure S3 Pairwise Meta-analysis of exercise on SBP

Study or Subgroup	Experimental Mean	Experimental SD	Experimental Total	Control Mean	Control SD	Control Total	Weight	Mean Difference IV, Random, 95% CI
Alexei2018	139	4.47	20	146	4.58	21	6.8%	-7.00 [-9.77, -4.23]
Anahita2018	125	9	12	120.8	8.4	12	4.5%	4.20 [-2.77, 11.17]
Arca2014	124	15	14	132.4	6.21	14	3.7%	-8.40 [-16.90, 0.10]
Arturo2011	115.97	3.12	12	123.93	14.48	12	3.8%	-7.96 [-16.34, 0.42]
Asako2012	100	15	11	110	13	11	2.6%	-10.00 [-21.73, 1.73]
Azadpour2017	122.08	3.58	12	129.67	3.77	12	6.7%	-7.59 [-10.53, -4.65]
Colado2009	123.5	11.4	15	132.9	11.2	10	3.5%	-9.40 [-18.43, -0.37]
Eun2019	136.42	13.79	34	133.2	19.3	13	2.7%	3.22 [-8.25, 14.69]
Figueroa2012	128.01	10.2	15	138	11	13	4.0%	-9.99 [-17.89, -2.09]
Gerage2013	120	7	15	126	8	14	5.3%	-6.00 [-11.49, -0.51]
Jeon2018	137	8.5	8	142	8.5	8	3.8%	-5.00 [-13.33, 3.33]
Jinkee2017	126.8	5	25	128.9	6.8	25	6.5%	-2.10 [-5.41, 1.21]
Masanori2012	136.98	10.8	13	128.3	12.5	13	3.5%	8.68 [-0.30, 17.66]
Nihal2012	108.12	8.68	24	113.09	13.27	19	4.5%	-4.97 [-11.87, 1.93]
Nishida2015	132.3	14.9	31	133.8	17.5	31	3.9%	-1.50 [-9.59, 6.59]
Pak2014	139.2	11.5	15	134.1	19.9	13	2.4%	5.10 [-7.18, 17.38]
Sattar2012	113.53	17.69	14	127.87	12.58	10	2.5%	-14.34 [-26.45, -2.23]
Tze2019	118.4	22.13	30	118.5	17.47	30	3.1%	-0.10 [-10.19, 9.99]
Won2017	132.5	12.02	10	146.11	7.65	10	3.6%	-13.61 [-22.44, -4.78]
Won2020	135.5	5	10	138.9	4.1	10	6.1%	-3.40 [-7.41, 0.61]
Wong2016	131	14.42	13	137	14.42	13	2.8%	-6.00 [-17.09, 5.09]
Wong2018	135	7.21	52	145	6.93	48	6.8%	-10.00 [-12.77, -7.23]
Woo 2016	138.76	5.98	25	152.4	1.41	25	6.9%	-13.64 [-16.05, -11.23]
**Total (95% CI)**			**430**			**387**	**100.0%**	**-5.65 [-7.98, -3.33]**

Heterogeneity: Tau² = 18.83; Chi² = 82.37, df = 22 (P < 0.00001); I² = 73%
Test for overall effect: Z = 4.76 (P < 0.00001)

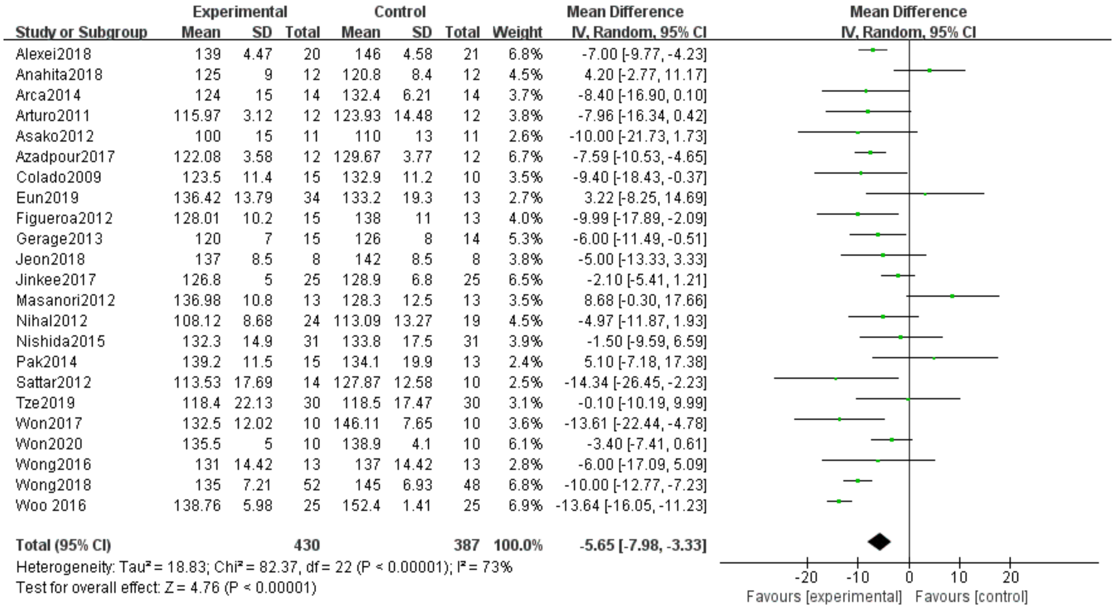

Figure S4 Pairwise Meta-analysis of exercise on DBP

Study or Subgroup	Experimental Mean	Experimental SD	Experimental Total	Control Mean	Control SD	Control Total	Weight	Mean Difference IV, Fixed, 95% CI
Alexei2018	86	4.47	20	89	4.58	21	2.3%	-3.00 [-5.77, -0.23]
Anahita2018	79.6	6.06	12	80.4	5.6	12	0.8%	-0.80 [-5.47, 3.87]
Arca2014	78.89	2.32	14	84.26	3.94	14	3.1%	-5.37 [-7.77, -2.97]
Arturo2011	67.31	9.98	12	72.14	9.87	12	0.3%	-4.83 [-12.77, 3.11]
Asako2012	62	7	11	69	6	11	0.6%	-7.00 [-12.45, -1.55]
Azadpour2017	80	0.43	12	82.83	0.72	12	78.4%	-2.83 [-3.30, -2.36]
Colado2009	77.9	7.4	15	81	8	10	0.5%	-3.10 [-9.31, 3.11]
Eun2019	78.91	8.5	34	76.3	10.4	13	0.4%	2.61 [-3.72, 8.94]
Figueroa2012	75	8	15	80	7	13	0.6%	-5.00 [-10.56, 0.56]
Gerage2013	80	6	15	82	5	14	1.1%	-2.00 [-6.01, 2.01]
Jeon2018	85	5.66	8	89	5.66	8	0.6%	-4.00 [-9.55, 1.55]
Jinkee2017	77.8	4.4	25	78.6	4.6	25	2.8%	-0.80 [-3.30, 1.70]
Masanori2012	81.8	9.1	13	76.8	12.5	13	0.2%	5.00 [-3.40, 13.40]
Nihal2012	70.2	8.64	24	73.8	7.56	19	0.8%	-3.60 [-8.45, 1.25]
Nishida2015	80.1	11.4	31	80.9	14.3	31	0.4%	-0.80 [-7.24, 5.64]
Pak2014	75.9	8.9	13	73.7	12.6	10	0.2%	2.20 [-6.99, 11.39]
Tze2019	77.6	12.54	30	74.9	12.1	30	0.5%	2.70 [-3.54, 8.94]
Won2017	84.41	9.08	10	95.4	6.32	10	0.4%	-10.99 [-17.85, -4.13]
Won2020	80	3.8	10	81.7	2.4	10	2.3%	-1.70 [-4.49, 1.09]
Wong2016	74	14.42	13	79	7.21	13	0.2%	-5.00 [-13.76, 3.76]
Wong2018	88	7.21	52	91	6.93	48	2.3%	-3.00 [-5.77, -0.23]
Woo 2016	84.56	1.26	25	90.52	9.2	25	1.3%	-5.96 [-9.60, -2.32]
**Total (95% CI)**			**414**			**374**	**100.0%**	**-2.85 [-3.27, -2.43]**

Heterogeneity: Chi² = 31.00, df = 21 (P = 0.07); I² = 32%
Test for overall effect: Z = 13.31 (P < 0.00001)

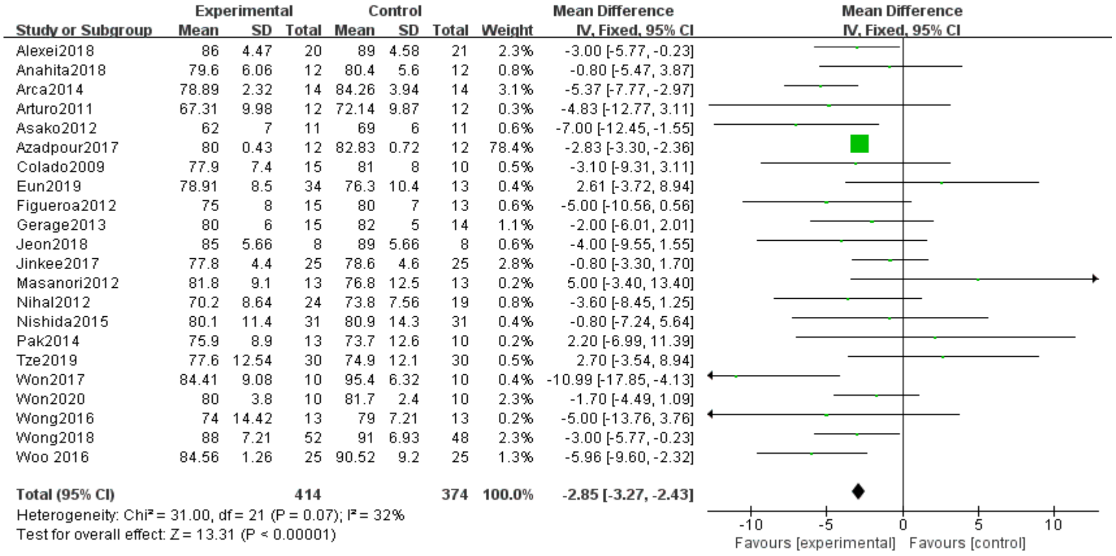

Figure S5 Pairwise Meta-analysis of exercise on TC

	Experimental			Control				Mean Difference	Mean Difference
Study or Subgroup	Mean	SD	Total	Mean	SD	Total	Weight	IV, Fixed, 95% CI	IV, Fixed, 95% CI
Anahita2018	181.6	25.6	12	198.3	22.6	12	3.8%	-16.70 [-36.02, 2.62]	
Arca2014	211	42.8	19	210	29.9	14	2.3%	1.00 [-23.81, 25.81]	
Asako2012	230.8	27.06	11	226.93	23.97	11	3.1%	3.87 [-17.49, 25.23]	
Cleiton2014	207.03	37.32	23	201.42	37.5	12	2.1%	5.61 [-20.52, 31.74]	
Colado2009	220.4	19.9	15	242.2	52.3	10	1.2%	-21.80 [-55.74, 12.14]	
HuiHe2022	154.64	27.06	15	145.75	17.78	14	5.2%	8.89 [-7.67, 25.45]	
Jeon2018	177	25.46	8	185	39.6	8	1.3%	-8.00 [-40.62, 24.62]	
Jinkee2017	184.8	10.2	25	187.6	9.5	25	47.8%	-2.80 [-8.26, 2.66]	
Kim2021	200.36	37.9	12	225.29	22.21	10	2.2%	-24.93 [-50.41, 0.55]	
Leandro2020	202.89	41.2	40	212.03	28.34	19	4.4%	-9.14 [-27.18, 8.90]	
Masanori2012	214.01	21.7	13	221.2	27.5	13	3.9%	-7.19 [-26.23, 11.85]	
Nihal2012	198.25	43.4	24	225	51.83	19	1.7%	-26.75 [-55.81, 2.31]	
Nishida2015	215.8	30	31	223.8	30.5	31	6.3%	-8.00 [-23.06, 7.06]	
Paolo2019	196.08	38.6	25	215.04	26.33	23	4.1%	-18.96 [-37.53, -0.39]	
Rodrigo2008	222.92	31.19	36	226.14	51.51	22	2.5%	-3.22 [-27.03, 20.59]	
Takeshima2002	219.6	33.94	15	227.2	25	15	3.1%	-7.60 [-28.93, 13.73]	
Tze2019	227.71	50.65	30	223.84	42.53	30	2.6%	3.87 [-19.80, 27.54]	
Zois2009	213	37.3	10	231.7	18.8	10	2.1%	-18.70 [-44.59, 7.19]	
**Total (95% CI)**			**364**			**298**	**100.0%**	**-5.23 [-9.00, -1.45]**	

Heterogeneity: Chi² = 15.99, df = 17 (P = 0.52); I² = 0%
Test for overall effect: Z = 2.71 (P = 0.007)

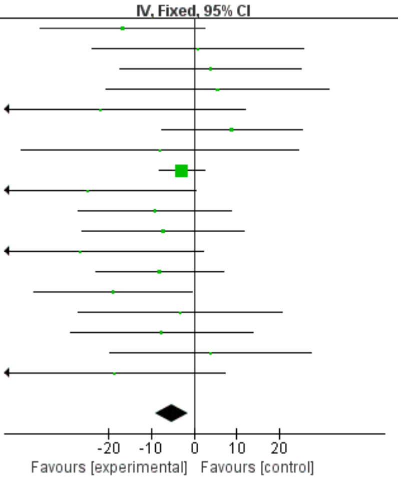

Figure S6 Pairwise Meta-analysis of exercise on TG

	Experimental			Control				Mean Difference	Mean Difference
Study or Subgroup	Mean	SD	Total	Mean	SD	Total	Weight	IV, Random, 95% CI	IV, Random, 95% CI
Anahita2018	135.6	15.8	12	187.6	21.5	12	7.0%	-52.00 [-67.10, -36.90]	
Zois2009	127.6	6.6	10	166.4	7.4	10	8.6%	-38.80 [-44.95, -32.65]	
Pak2014	122.27	23.92	13	150.62	24.92	10	6.0%	-28.35 [-48.54, -8.16]	
Takeshima2002	86.1	27.8	15	113.9	47.8	15	4.6%	-27.80 [-55.78, 0.18]	
Tze2019	92.14	7.5	30	119.61	5.09	30	8.9%	-27.47 [-30.71, -24.23]	
Asako2012	93.92	42.53	11	121.38	51.39	11	3.1%	-27.46 [-66.88, 11.96]	
Nihal2012	149.58	53.64	24	160.42	46.17	19	4.3%	-10.84 [-40.70, 19.02]	
Jeon2018	117	53.74	8	127	42.43	8	2.4%	-10.00 [-57.45, 37.45]	
Woo 2016	165.52	4.34	25	175.52	3.42	25	9.0%	-10.00 [-12.17, -7.83]	
Nishida2015	95.97	42.5	31	103.5	34	31	6.2%	-7.53 [-26.69, 11.63]	
Masanori2012	100.1	53.9	13	107.21	41.6	13	3.3%	-7.11 [-44.12, 29.90]	
Leandro2020	125.64	58.51	40	130.31	57.35	19	4.0%	-4.67 [-36.19, 26.85]	
Jinkee2017	115.2	10.7	25	116.2	10.4	25	8.7%	-1.00 [-6.85, 4.85]	
Rodrigo2008	96.08	39.12	36	87.05	32.76	22	6.3%	9.03 [-9.70, 27.76]	
Arca2014	146	67.1	19	133	56.8	14	2.8%	13.00 [-29.37, 55.37]	
Paolo2019	125.16	55.76	25	111.82	39.07	23	4.7%	13.34 [-13.73, 40.41]	
Colado2009	97.7	36.3	15	84.1	36.9	10	4.4%	13.60 [-15.73, 42.93]	
HuiHe2022	80.63	38.1	15	58.48	20.38	15	5.7%	22.15 [0.28, 44.02]	
**Total (95% CI)**			**367**			**312**	**100.0%**	**-12.46 [-20.97, -3.95]**	

Heterogeneity: Tau² = 208.13; Chi² = 210.26, df = 17 (P < 0.00001); I² = 92%
Test for overall effect: Z = 2.87 (P = 0.004)

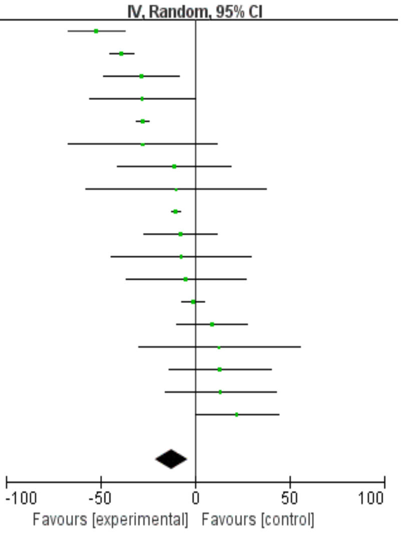

Figure S7 Pairwise Meta-analysis of exercise on LDL-C

	Experimental			Control				Mean Difference	Mean Difference
Study or Subgroup	Mean	SD	Total	Mean	SD	Total	Weight	IV, Fixed, 95% CI	IV, Fixed, 95% CI
Anahita2018	172.7	12.5	12	172.7	9.5	12	15.1%	0.00 [-8.88, 8.88]	
Arca2014	131	40.3	19	131	23.7	14	2.5%	0.00 [-21.97, 21.97]	
Asako2012	141.8	23.9	11	134.9	17.01	11	4.0%	6.90 [-10.44, 24.24]	
Cleiton2014	124.26	26.9	23	121.01	18.17	12	5.2%	3.25 [-11.80, 18.30]	
Colado2009	133.6	24.6	15	159.2	47.3	10	1.2%	-25.60 [-57.45, 6.25]	
HuiHe2022	90.08	22.42	15	90.85	25.52	15	4.0%	-0.77 [-17.96, 16.42]	
Jinkee2017	98.4	9.2	25	102.4	11.8	25	34.5%	-4.00 [-9.87, 1.87]	
Kim2021	115.34	33.52	12	122.54	28.65	10	1.8%	-7.20 [-33.18, 18.78]	
Leandro2020	118.5	32.14	40	131.32	43.05	19	2.5%	-12.82 [-34.59, 8.95]	
Masanori2012	128.2	22	13	131.32	26.1	13	3.4%	-3.12 [-21.68, 15.44]	
Nihal2012	126.04	32.09	24	136.47	46.05	19	2.0%	-10.43 [-34.79, 13.93]	
Nishida2015	131.16	24.6	31	134.1	26.8	31	7.2%	-2.94 [-15.75, 9.87]	
Pak2014	194.07	31.32	13	196.01	48.33	10	1.0%	-1.94 [-36.40, 32.52]	
Paolo2019	102.81	30.69	25	133.81	23.72	23	5.0%	-31.00 [-46.45, -15.55]	
Rodrigo2008	134.71	28.68	36	140.57	47.87	22	2.4%	-5.86 [-27.95, 16.23]	
Takeshima2002	128.6	39.1	15	121.8	2.1	15	3.0%	6.80 [-13.02, 26.62]	
Tze2019	148.84	52.96	30	142.66	40.21	30	2.1%	6.18 [-17.61, 29.97]	
Zois2009	133.9	21.7	10	159.9	23	10	3.1%	-26.00 [-45.60, -6.40]	
**Total (95% CI)**			**369**			**301**	**100.0%**	**-4.42 [-7.86, -0.97]**	

Heterogeneity: Chi² = 24.61, df = 17 (P = 0.10); I² = 31%
Test for overall effect: Z = 2.51 (P = 0.01)

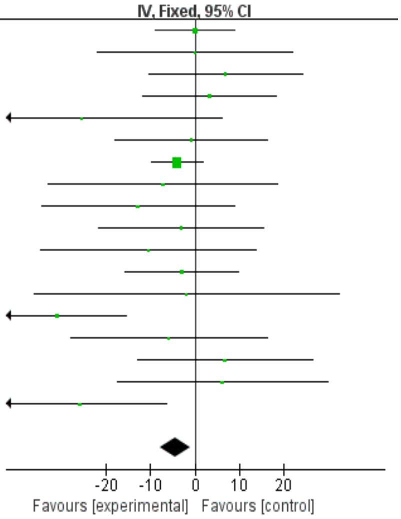

Figure S8 Pairwise Meta-analysis of exercise on HDL-C

Study or Subgroup	Experimental Mean	Experimental SD	Experimental Total	Control Mean	Control SD	Control Total	Weight	Mean Difference IV, Random, 95% CI
Anahita2018	42.9	7.1	12	33.6	3.5	12	7.0%	9.30 [4.82, 13.78]
Arca2014	51	15.4	19	51	11.2	14	4.4%	0.00 [-9.08, 9.08]
Asako2012	71.91	11.99	11	64.949	13.144	11	3.8%	6.96 [-3.55, 17.47]
Cleiton2014	59.25	18.93	23	63.016	8.892	12	4.4%	-3.77 [-12.99, 5.46]
Colado2009	68.1	12	15	73.6	9.9	10	4.6%	-5.50 [-14.13, 3.13]
HuiHe2022	45.62	10.44	15	46.78	5.8	15	6.1%	-1.16 [-7.20, 4.88]
Jinkee2017	50.3	5.9	25	49.1	5.5	25	7.7%	1.20 [-1.96, 4.36]
Kim2021	41.45	21.32	12	47.82	27.56	10	1.4%	-6.37 [-27.28, 14.54]
Leandro2020	58.52	21.31	40	52.2	25.56	19	2.9%	6.32 [-6.94, 19.58]
Masanori2012	65.7	7.5	13	69.7	9.5	13	5.7%	-4.00 [-10.58, 2.58]
Nihal2012	52.08	10.39	24	57.28	9.08	19	6.2%	-5.20 [-11.03, 0.63]
Nishida2015	65.2	8.7	31	71.1	10	31	6.9%	-5.90 [-10.57, -1.23]
Pak2014	44.85	12.37	13	45.62	18.56	10	2.8%	-0.77 [-14.09, 12.55]
Paolo2019	60.12	17.04	25	58.34	16.8	23	4.2%	1.78 [-7.80, 11.36]
Rodrigo2008	69.86	10.94	36	69.24	8.41	22	6.7%	0.62 [-4.39, 5.63]
Takeshima2002	62.3	15.5	15	67.4	17.1	15	3.4%	-5.10 [-16.78, 6.58]
Tze2019	64.56	17.01	30	56.44	14.69	30	4.9%	8.12 [0.08, 16.16]
Woo 2016	43.32	2.34	25	33.64	2	25	8.5%	9.68 [8.47, 10.89]
Zois2009	50.3	1.3	10	42.6	1.4	10	8.5%	7.70 [6.52, 8.88]
**Total (95% CI)**			**394**			**326**	**100.0%**	**1.44 [-1.30, 4.18]**

Heterogeneity: Tau² = 22.76; Chi² = 127.86, df = 18 (P < 0.00001); I² = 86%
Test for overall effect: Z = 1.03 (P = 0.30)

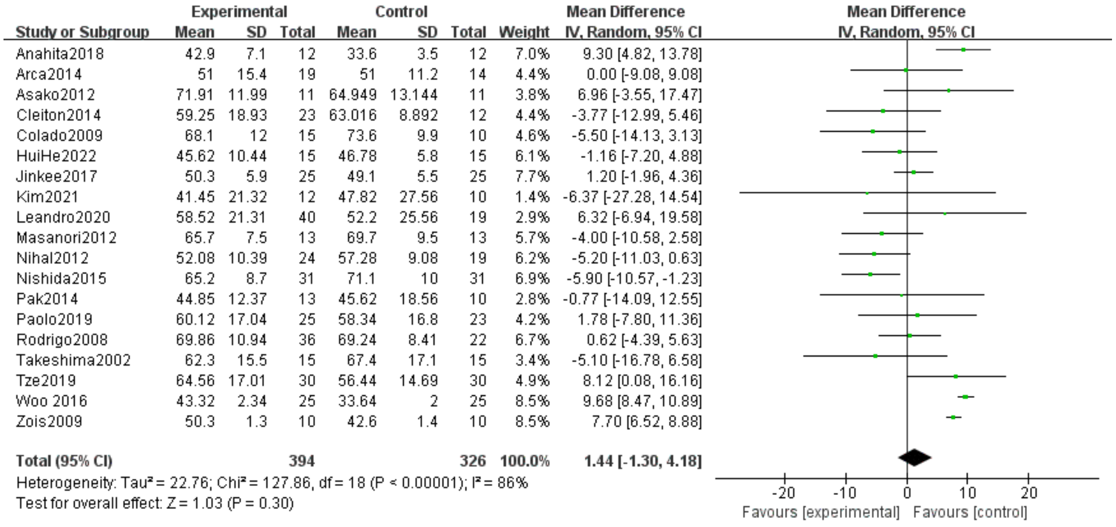

Figure S9 Cumulative ranking probability plots for SBP

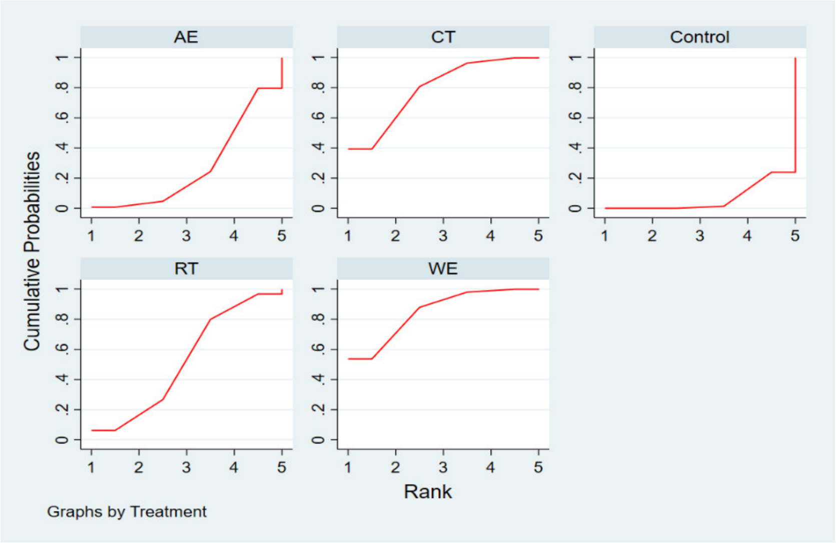

Figure S10 Cumulative ranking probability plots for DBP

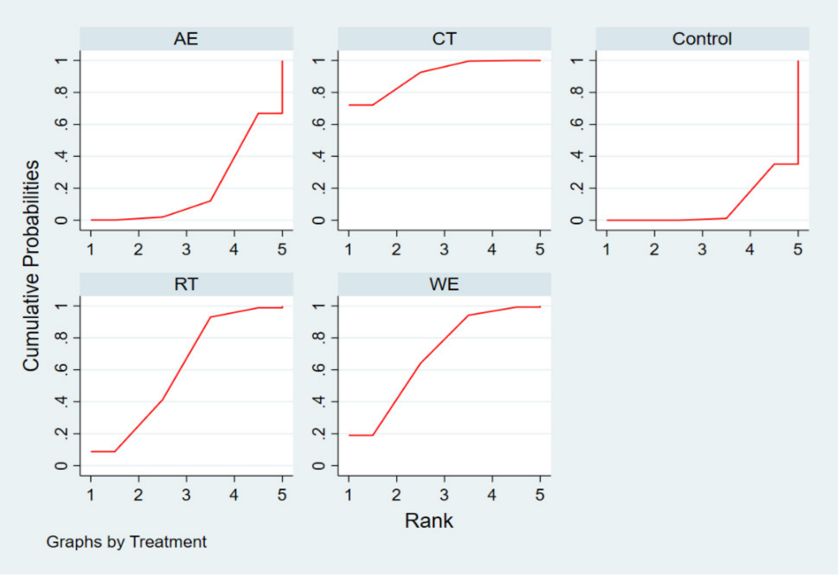

Figure S11 Cumulative ranking probability plots for TC

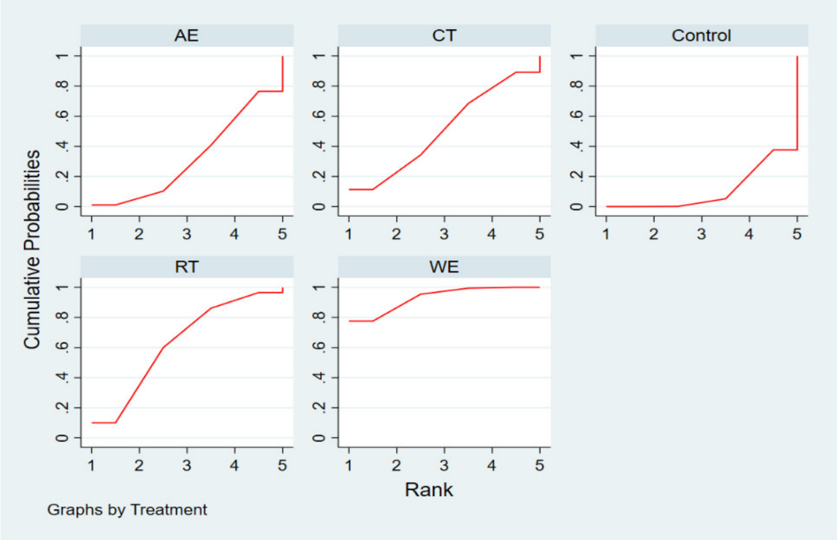

Figure S12 Cumulative ranking probability plots for TG

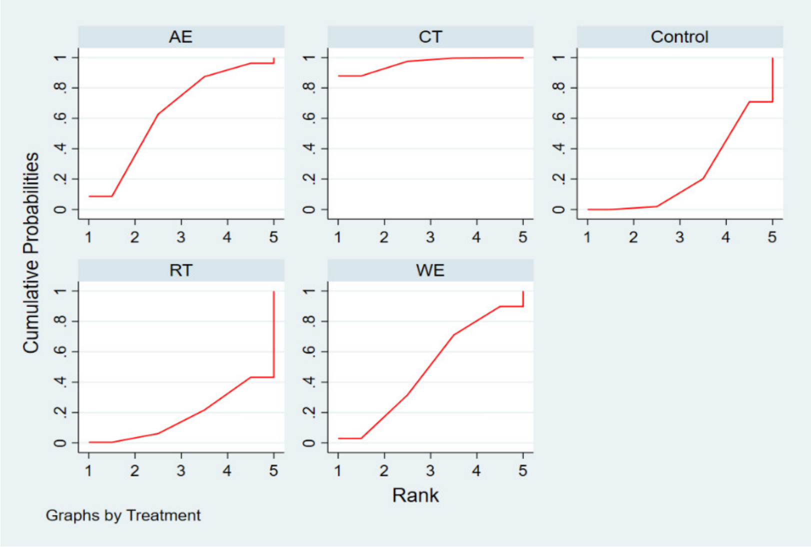

Figure S13 Cumulative ranking probability plots for LDL-C

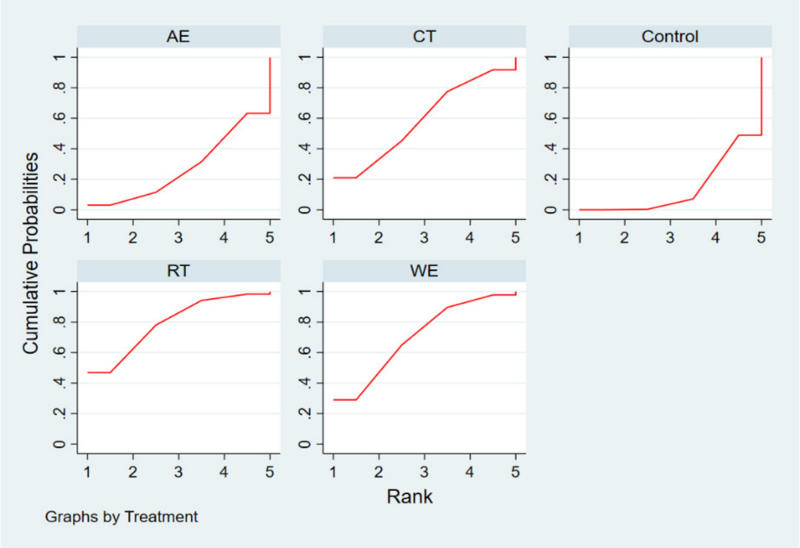

Figure S14 Cumulative ranking probability plots for HDL-C

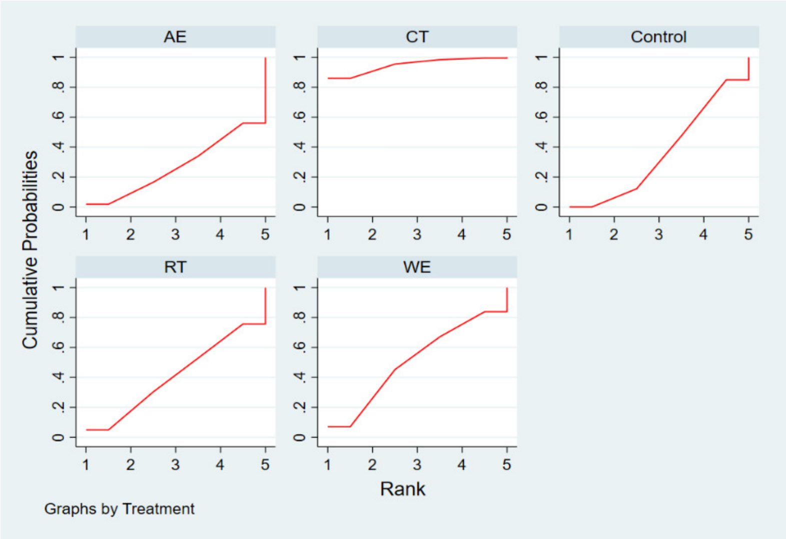

Figure S15 Network meta-analysis funnel plots for SBP

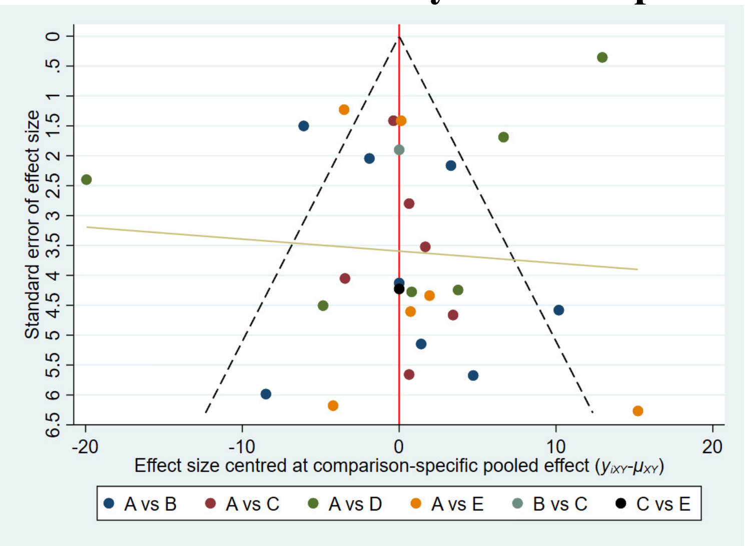

Figure S16 Network meta-analysis funnel plots for DBP

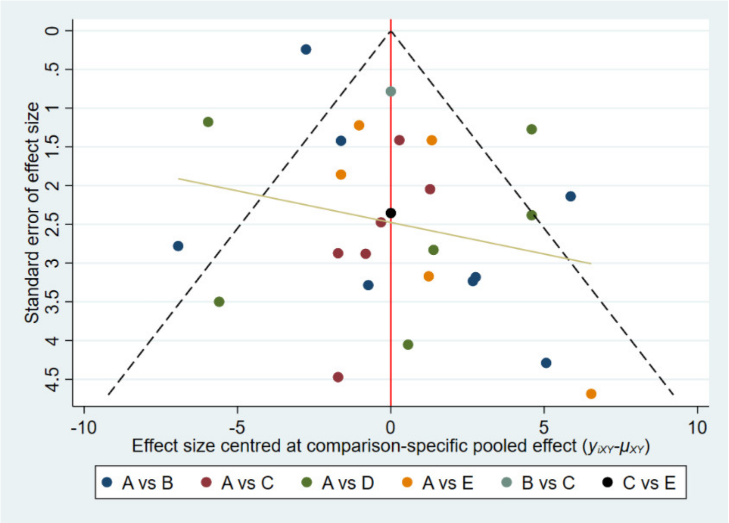

Figure S17 Network meta-analysis funnel plots for TC

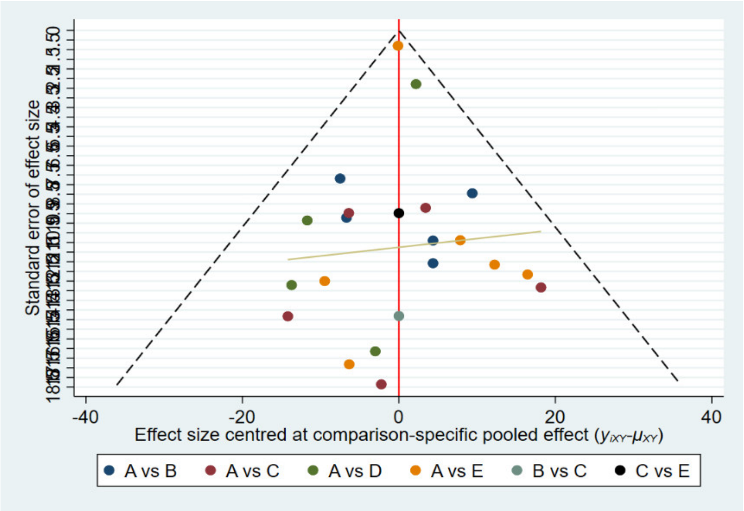

Figure S18 Network meta-analysis funnel plots for TG

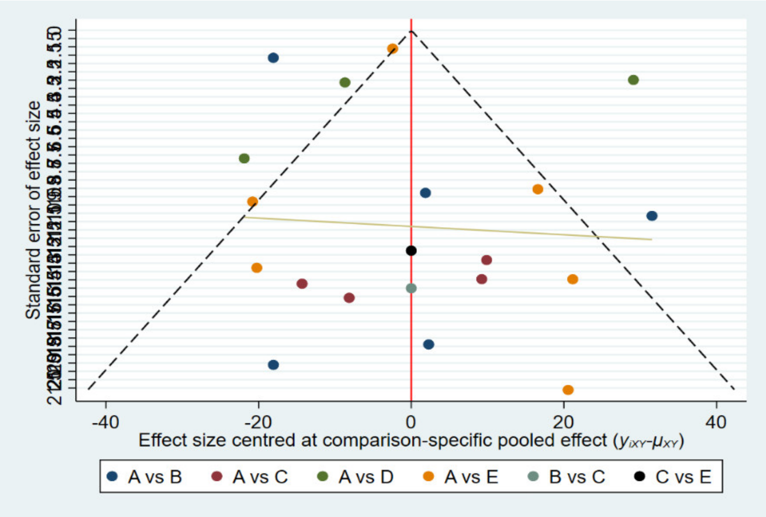

Figure S19 Network meta-analysis funnel plots for LDL-C

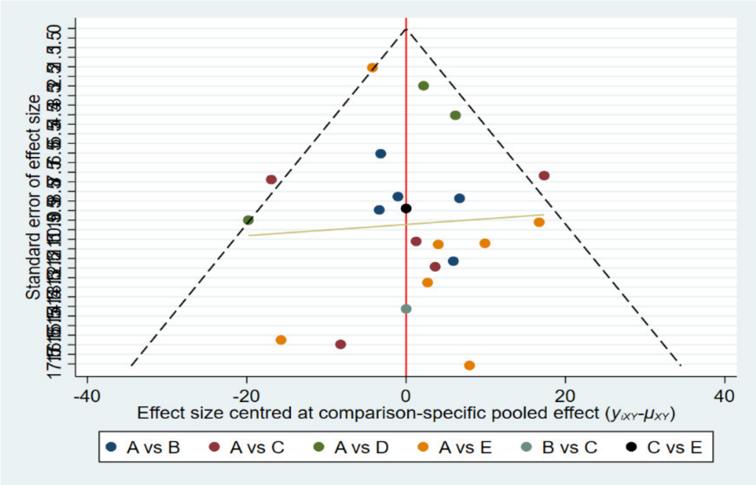

Figure S20 Network meta-analysis funnel plots for HDL-C

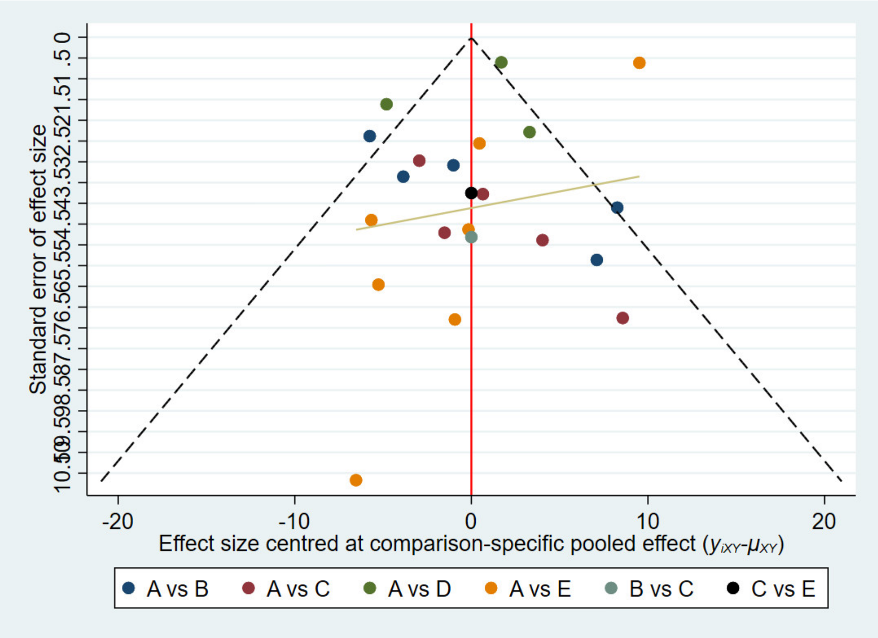

Supplement: Supplementary file 1 [file ijerph-19-12074-s001.zip › ijerph-1909360-supplementary.pdf]
